# Nutritional Assessment of Children and Adolescents with Atypical Anorexia Nervosa: A Preliminary Longitudinal Investigation Using the 24-h Dietary Recall

**DOI:** 10.3390/children11040427

**Published:** 2024-04-03

**Authors:** Beatrice Valeriani, Jacopo Pruccoli, Francesca Chiavarino, Maria Letizia Petio, Antonia Parmeggiani

**Affiliations:** 1 IRCCS-Azienda Ospedaliero-Universitaria Di Bologna, Clinical Nutrition and Metabolism Unit, 40138 Bologna, Italy; beatrice.valeriani@aosp.bo.it; 2 IRCCS Istituto delle Scienze Neurologiche di Bologna, Regional Center for Feeding and Eating Disorders in the Developmental Age, Child Neurology and Psychiatry Unit, 40138 Bologna, Italy; jacopo.pruccoli2@unibo.it (J.P.); francesca.chiavarino@studio.unibo.it (F.C.); 3 Department of Medical and Surgical Sciences (DIMEC), University of Bologna, 40138 Bologna, Italy; marialetizia.petio2@studio.unibo.it

**Keywords:** Atypical Anorexia Nervosa, children and adolescents, 24 h dietary recall, nutrition, energy intake, BMI

## Abstract

Background: Atypical Anorexia Nervosa (AAN) is a Feeding and Eating Disorder characterized by fear of gaining weight and body image disturbance, in the absence of significantly low body weight. AAN may present specific clinical and psychopathological features. Nonetheless, the literature lacks data concerning the nutritional characteristics and body composition of children and adolescents with AAN and their variation over time. Methods: Case series, including 17 children and adolescents with AAN. All the patients were assessed at the first evaluation (T0) with a standardized dietary assessment (24 h Dietary Recall, 24 hDR). Nutritional data were compared with European dietary reference values (DRVs). Body composition parameters (weight, fat mass, fat-free mass) and their changes over time at two (T1) and six (T2) months were collected as well, using a Bioelectrical impedance analysis (Wunder WBA300 with four poles and foot contact; impedance frequency 50 kHz 500 μA; impedance measurement range 200~1000 Ω/0.1 Ω). Results: The included individuals presented eating behaviors oriented towards significantly low daily energy intake (*p* < 0.001) compared with DRVs set by the European Food Safety Authority (EFSA) (with low carbohydrates and fats), and increased proteins (*p* < 0.001). A longer latency before observation (illness duration before observation) correlated with a negative change in weight. Body composition parameters were described, with no significant changes across the six-month outpatient assessment. Discussion: This is the first research to systematically assess the body composition and nutritional features of a group of individuals with AAN in the developmental age. Further research should assess the effect of targeted treatment interventions on body composition and nutritional features.

## 1. Introduction

### 1.1. Classification and Definition

Prior to the release of the Fifth Edition of the Diagnostic and Statistical Manual of Mental Disorders (DSM-5) [1], individuals seeking treatment for Feeding and Eating Disorders (FED) at specialized programs, who did not meet the criteria for Anorexia Nervosa (AN) or Bulimia Nervosa, were often diagnosed with “Eating Disorder Not Otherwise Specified” (EDNOS) [2]. EDNOS, the most frequently diagnosed FED, encompassed a heterogeneous range of presentations, providing clinicians with limited diagnostic insights and posing challenges for effective treatment [3]. Subsequent revisions to the DSM-5 criteria aimed to enhance clarity by eliminating the EDNOS diagnosis and introducing a new category termed Other Specified Feeding or Eating Disorder (OSFED). OSFED comprises five distinct disorders, including “Atypical Anorexia Nervosa” (AAN), Bulimia Nervosa of low frequency and/or limited duration, Binge Eating Disorder of low frequency and/or limited duration, Purging Disorder, and Night Eating Syndrome [1].

AAN, a novel category in DSM-5, delineates individuals meeting criteria B (intense fear of gaining weight or interference with weight gain) and C (body image disturbance) for AN, without criterion A (low body weight), despite significant weight loss [1]. This condition can manifest in individuals with normal weight, overweight, or obesity initially, with subsequent weight loss and restrictive behaviors leading to malnourishment [3]. The prevalence of adolescent AAN has been reported as 2.8% in a community sample [4] and 16.4% in a clinical sample of adolescents with FED [5].

Adolescents diagnosed with AAN demonstrate higher rates of suicidality compared to peers without eating disorders [4] but exhibit similar rates of self-harm and suicidality compared to peers with AN [5]. Research suggests that adolescents with AAN may display more severe eating disorder psychopathology [5,6,7,8] and comparable levels of poor self-esteem [5] compared to adolescents with AN. While preliminary case-report data exist on the adoption of nutritional guidelines for these patients [9], further comprehensive data are imperative for a more thorough understanding.

### 1.2. The Nutritional Assessments of Individuals with FED

A key clinical feature across different FEDs is represented by a reduction or a loss of control of food intake [1]. To assess energy intake, a validated tool is represented by the 24 h dietary recall (24 hDR). The 24 hDR involves an in-depth interview where the patient describes the intake of the previous 24 h. This tool can be administered quickly, but the presence of a trained interviewer is required. The methodology behind the 24 hDR relies on the individual memory of the patients, thus being prone to potential omissions.

Recollections of past dietary intake can be susceptible to influence from psychopathological factors, particularly evident among individuals with a restrictive FED. A consistent observation in this group is an inclination to under-report energy intake. Those reporting low energy intakes tend to indicate a higher percentage of energy derived from protein and a lower percentage from fats and sugars [10]. Despite these challenges, the 24 h Dietary Recall (24 hDR) remains widely employed in dietary surveys and research due to its relatively low burden on patients [11].

An alternative validated method for estimating current dietary intake is through the use of Dietary Records (DRs). A DR involves an open-ended, prospective dietary assessment, prompting individuals to report all foods and beverages consumed within a specific timeframe [12,13]. When implemented with proper procedures, DRs can exhibit high validity and considerable precision. However, limitations exist, including a tendency for subjects to report socially desirable food consumption. Additionally, challenges related to the respondent burden may arise, with some individuals struggling to document consumed items or describe portion sizes. Consequently, the quality of completed diet records diminishes as the observed period lengthens. On a positive note, a DR offers a realistic registration of actual food and beverage consumption, mitigating issues associated with caloric omissions resulting from memory lapses [13].

Scarce data concerning the nutritional features of AAN are available. Hypophosphatemia, a marker of Refeeding Syndrome, has been reported with a considerable frequency (41%) among a group of 171 adolescents with AN or AAN [14]. In a further study comparing subjects with AAN to those with AN, AAN was found to have a higher frequency of premorbid overweight or obesity, with a higher weight loss in longer time frames [5]. Individuals with AAN may show greater Z-scores in bone mineral content and density, as well as fat mass index and lean Body Mass Index when compared to AN [15].

### 1.3. Aim of the Study

The study of metabolic factors involved in the clinical pictures of AAN has been reported among the research priorities for the study of AAN in a recent Delphi study [16]. Despite this evidence and the increasing prevalence of AAN during the recent SARS-CoV-2 pandemic [17], the literature still lacks data concerning the nutritional status of children and adolescents with AAN.

In this study, we aim to investigate the clinical and nutritional characteristics of a group of children and adolescents diagnosed with AAN, who accessed a third-level Center for a FED in the developmental age, as systematically and longitudinally assessed via a 24 hDR and a Bioelectrical impedance analysis (BIA).

## 2. Materials and Methods

### 2.1. Study Design and Participants

This study is a case series of 17 patients assessed between 1 January 2022 and 30 November 2022 at the Regional Centre for Feeding and Eating Disorders in children and adolescents in Bologna, Italy. Inclusion criteria were (a) a diagnosis of AAN according to DSM-5 [1]; and (b) the compilation of a 24 h dietary recall at first assessment, usually utilized during outpatient visits. Both children and adolescents were included; an arbitrary cutoff of 14 years of age to define childhood/adolescence was adopted, referring to the descriptions of two relevant studies addressing FED in the developmental age [18,19].

Given the developmental age of the included patients, according to the DSM-5 criteria, the weight threshold to diagnose AAN (instead of “classic” AN) was established using percentiles and growth charts instead of a fixed value. Instead of relying solely on Body Mass Index (BMI), this study opted for the percentage of a normal BMI for age and gender (%BMI). The utilization of %BMI is recommended by the Junior MARSIPAN report, which addresses the management of severely ill patients under 18 with Anorexia Nervosa. The %BMI is calculated as (BMI/median BMI for age and gender × 100) [20,21]. Reference values from the World Health Organization BMI-for-age charts for girls and boys were employed in this study [22]. A %BMI threshold of 85 was established for diagnosing AAN, aligning with established international literature in this field [14,16,17].

In this case series, explicit informed consent was obtained from all participating families and patients for the utilization of their data for research purposes, adhering to the hospital’s ethics protocol. The study was conducted in compliance with the principles outlined in the Declaration of Helsinki.

### 2.2. Assessment Methods

For all the included patients, data were collected at T0 (first assessment), T1 (second assessment), and T2 (third assessment). The evaluations were scheduled according to the clinical necessities of the included patients; thus, no pre-structured timeline for assessments was programmed.

At all the considered assessments (T0, T1, T2), the following measures were collected for the included patients:

Anthropometric measures were taken at baseline, where weight was measured using a calibrated digital scale (Wunder WBA) with participants not wearing clothing. Height without shoes was measured using a stadiometer. The recorded data included weight, height, BMI (Body Mass Index), and %BMI.

Body composition: Concurrently with the weight measurement, a BIA was performed using Wunder WBA300 (measuring system Bioelectrical impedance analysis with four poles and foot contact; impedance frequency 50 kHz 500 μA; impedance measurement range 200~1000 Ω/0.1 Ω). Fat mass percentage (%FM) and fat-free mass (FFM) were assessed.

BIA represents a widely employed method for gauging body composition across various clinical contexts, including cancer, obesity, sarcopenia, and in the elderly. The market offers a variety of BIA devices, categorized by electrical frequency into single-frequency (SF-BIA) and multifrequency (MF-BIA). Generally, both SF-BIA and MF-BIA devices exhibit a high precision level, typically with a 1–2% variability between repeated measures [23]. The accuracy and precision of BIA devices depend on various factors, including patient-related aspects such as the degree of adiposity, fluid and electrolyte status, and skin temperature. Environmental factors like ambient temperature, proximity to metal surfaces, and electronic devices also play a role. Additionally, the reliability of BIA results is influenced by assumptions related to prediction methods (SF-BIA or MF-BIA), instrumentation factors, and variations in measurement protocols. On a positive note, BIA is praised for its portability, cost-effectiveness, quick and noninvasive nature, simplicity, reproducibility, and safety for repeated measures. However, there are drawbacks, including its indirect nature, reliance on hydration status (with fat-free mass hydration fixed at 73%), and the need for specific equations tailored to each population [23].

Demographic (age, gender), clinical (symptoms of FED and comorbidities), and treatment (nutritional and pharmacological interventions) variables were collected as well.

Moreover, at the moment of the first assessment (T0), a dietician specialized in FEDs in the developmental age administered a 24 hDR. The 24 h Dietary Recall (24 hDR) functions as a subjective, face-to-face (or telephonic) interview method [24]. It necessitates the patient to provide both quantitative and qualitative details regarding the foods and beverages consumed within the 24 h preceding the interview. Comprehensive information, including types, characteristics, quantity, preparation methods, brand details, dressings, places of consumption, and any potential supplements, is expected to be reported. In order to gather these data, the interviewer may utilize either a pre-structured or open questionnaire, sometimes supplemented with visual aids such as pictures, photographic examples, and recipe ingredients. The administration of a 24 hDR typically requires 20 to 30 min [24].

### 2.3. Nutritional Assessment

The caloric intake was reported as total values in kcal/day, and percentages of single macronutrients (carbohydrates, fat, proteins) representing the total percentage of energy intake (%En). Subsequently, the Nutrient Adequacy Ratio (NAR) was computed, representing an individual’s nutrient intake as a percentage (capped at 100%) of the recommended allowance for that nutrient based on the patient’s sex and age (Reference Intake, RI). For these analyses, the dietary reference values (DRVs) established by the European Food Safety Authority (EFSA), standardized for age and sex, were employed as a benchmark [25].

For fat and carbohydrates, the EFSA DRVs for children and adolescents provide standardized values for Reference Intake (RI). Since the European DRVs do not present RI values for proteins, following [26], protein RI was obtained by deducting carbohydrate and fat RIs from total RI.

Regarding proteins, the European DRVs for children and adolescents include the Average Requirement (AR) and Population Reference Intake (PRI). In this context, the protein intake of the included patients was compared to these values. For instance, the AR values were as follows: 0.71 g/kg body weight per day for 13-year-old girls, 0.7 g/kg body weight per day for 14-year-old girls, 0.69 g/kg body weight per day for 15-year-old girls, 0.68 g/kg body weight per day for 16-year-old girls, and 0.67 g/kg body weight per day for 17-year-old girls. Similarly, the PRI values were: 0.88 g/kg body weight per day for 13-year-old girls, 0.87 g/kg body weight per day for 14-year-old girls, 0.85 g/kg body weight per day for 15-year-old girls, 0.84 g/kg body weight per day for 16-year-old girls, and 0.83 g/kg body weight per day for 17-year-old girls.

### 2.4. Statistical Analysis

Descriptive statistics were provided for the full sample. Continuous variables were reported using means and standardized deviations, while categorical variables were reported as total numbers and percentages. The values for the macronutrient intakes as obtained via the 24 hDR were descriptively compared to the ESFA DRVs [25]. Then, to assess potential correlations between changes occurring in weight measures (%BMI) and the latency before the first clinical observation, a bivariate correlation (Spearman’s rho) was run. Changes in anthropometric variables across 3 time points were assessed with a Repeated Measures ANOVA. To assess the RI and the NAR corresponding to each macronutrient, percentages were used. To compare the reported protein intake to each patient’s AR and PRI, t-tests were used. The significance level for the analyses was established at 0.05, and all tests conducted were two-tailed. Normality of data distribution and homogeneity of variance were assessed using Shapiro–Wilk’s and Levene’s tests, respectively. The statistical analyses were carried out using JASP version 16.4 for Windows.

## 3. Results

### 3.1. Demographic and Clinical Variables

We initially enrolled, on first access, 21 normal-weighted patients with FEDs. A total of 17 fulfilled the study inclusion criteria. Reasons for exclusion (n = 4) were, at the second evaluation, a primary diagnosis of BN (n = 2), typical AN (n = 1), or dropout of treatment (n = 1). The collected data included in the study were observed at the first outpatient access and the second assessment, distanced 52.2 ± 21.1 days, and at the third assessment, which occurred six months later.

The 17 patients (Table 1) with a diagnosis of AAN (F = 100%) had a mean age at the admission of 15.8 ± 1.3 years, ranging from 13.3 to 17.9 years.

The patients arrived at their first consultation with a history of untreated illness of 15.2 ± 7.7 months. In that period, the weight loss from the onset of the disease was 6.0 ± 5.7 kg. Three patients (corresponding to 17.6%) also presented hypothalamic amenorrhea.

We highlighted a psychiatric comorbidity in 64.7% of the patients (n = 11). Anxiety disorder is the most represented associated comorbidity.

After the first access, pharmacological treatment was started in eight patients (47.1%). Fluoxetine was the treatment majorly prescribed due to depressive symptoms and reported binges.

### 3.2. Anthropometric Variations (Body Composition) over Time

**Figure 1:**
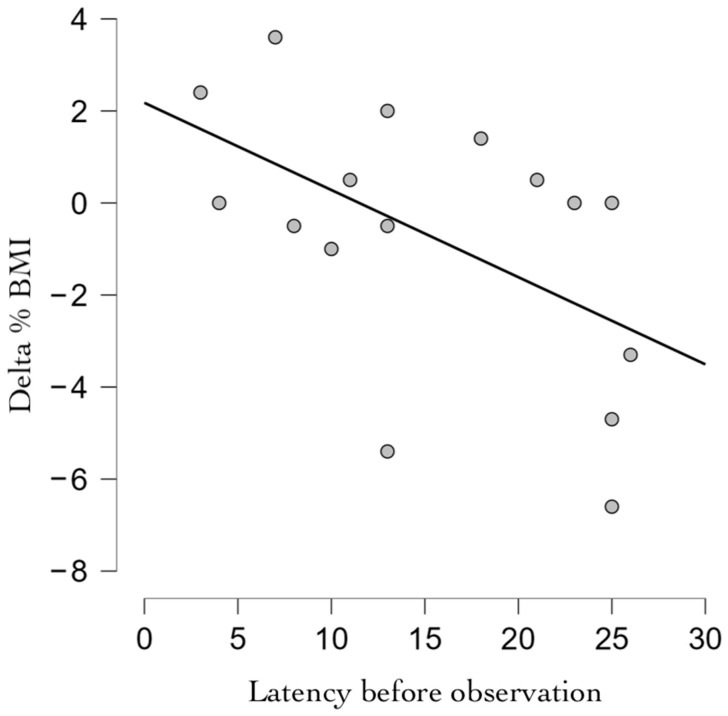
Correlation between %BMI change between admission and second evaluation, and latency before the observation. Abbreviations: %BMI: percentual Body Mass Index.

The assessed BMI was 20.4 ± 2.0 kg/m^2^ at the first evaluation (T0) and 20.0 ± 1.8 kg/m^2^ at the second evaluation (T1), with a mean difference of −0.15 kg/m^2^. The assessed %BMI was 99.5% ± 9.8 at T0, and 97.7% ± 8.6 at T1, with a mean difference of −0.7%. A longer latency before observation (illness duration before observation) was correlated with a negative change in %BMI (r = −0.527, *p* = 0.036), as documented in Figure 1.

Weight over time showed a variation between the onset (59.7 ± 7.6) and the first ambulatory access (T0); in the following assessments (T1, T2) substantial stability values were documented (Table 2).

### 3.3. Nutritional Assessment

The nutritional assessment (Table 3) was carried out through 24 hDR. From the collected data, we evaluated the total energy intake, equivalent to 1122 ± 180 kcal. This was significantly lower (*p* < 0.001) that the AR provided by the DRVs 2502 ± 56.3 kcal. The composition of the macronutrients was: proteins 22.9 ± 5.0%; fat 28.6 ± 5.0%; carbohydrates 47.8 ± 5.0%.

The median %En from total fats was close to the central value of the RI range (20–35%En). Contrariwise, the median %En from available carbohydrates was closer to the lower limit of the recommendations (45–60%).

**Figure 2:**
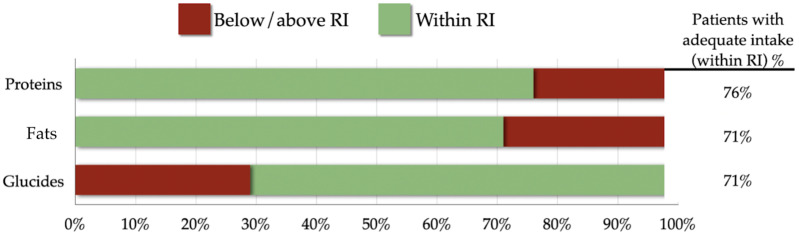
Nutritional adequacy of macronutrients in relation to the reference intake. The Nutrient Adequacy Ratio (NAR) was determined based on the Reference Intake (RI) range. Patients with intakes equal to the cut-off values were deemed adequate for that particular nutrient. Abbreviations: SD: standard deviation; AR: Average Requirement; RI: Reference Intake; PRI: Population Reference Intake; %En: percentage of Energy Intake.

Overall, the number of girls whose diet fell within the limits of the reference intakes was 76% for proteins, 71% for fat, and 71% for carbohydrates, respectively (Figure 2). On the contrary, 24% and 29% of the patients assumed protein and lipid intakes, respectively, higher than the adequate intake (AI), while 29% of them assumed fewer carbohydrates than recommended.

**Figure 3:**
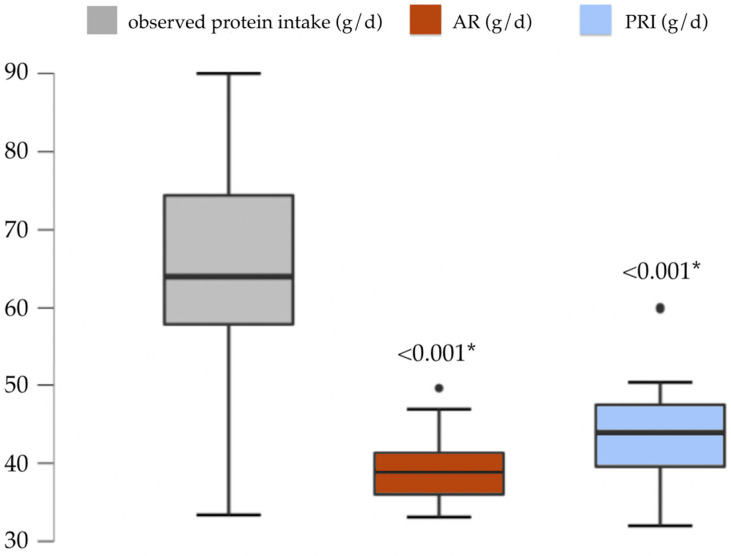
Box-and-whisker plots were utilized to compare the observed protein intake of our patients with their protein dietary reference values. In each box-and-whisker plot, the bottom and top edges of the box denote the 25th and 75th centile, representing the interquartile range. The line within each box signifies the median. The ends of the bottom and top whiskers indicate the minimum and maximum values, while circles represent outliers in the dataset. Asterisk (*) indicates statistical significance. Abbreviations: AR: Average Requirement; PRI: Population Reference Intake.

The observed distribution of protein intake, as illustrated in Figure 3, exhibited a moderate overlap with the corresponding required intakes. Statistical analysis, specifically the two-sample test for observed versus AR intake and observed versus PRI intake, yielded *p*-values of less than 0.001, indicating a significant difference between the observed and required intakes. Corresponding summary statistics were equal to observed intakes: 64.0 g/day (13.5–57.5) vs. AR intake: 37.5 ± 4.2 g/day, and PRI intake: 46.7 ± 5.0 g/day.

## 4. Discussion

AAN represents a recent clinical entity classified in DSM-5 among Other Specified Feeding and Eating Disorders. To date, psychological and clinical aspects have been discussed in the few studies which dealt with AAN, whereas the current literature presents scarce evidence focusing on diet and eating behavior in AAN.

In our case series, at present the largest sample reported, we describe preliminary results regarding the nutritional features and body composition of 17 patients diagnosed with AAN at a developmental age.

Garber and colleagues highlighted that adolescents who experienced a greater amount, rate, or duration of weight loss had significantly worse medical and nutritional status, independent of the admission weight [6]. These data were taken from evidence in other studies [7,14] which highlighted how, in patients diagnosed with restrictive FED, absolute weight, weight suppression (the difference between highest weight and presentation weight), and the rapidity of weight loss were better predictors than admission weight of many physical complications.

In our sample, longer latencies before admission were correlated with worse T1-T0 outcomes: in particular from the detection of anthropometric parameters, a significant negative correlation was seen between the waiting time between the onset of symptoms and the first outpatient access and the difference between the BMI at T0 and the BMI at the first outpatient visit T1. In our study, patients with a longer waiting time presented worse BMI improvements. These data are corroborated by prior studies indicating that individuals maintaining a healthy weight are less likely to be diagnosed with an eating disorder and are more likely to receive timely care [27]. A recent position paper from the Society for Adolescent Health and Medicine emphasized the importance of considering both the percentage of Body Mass Index (%BMI) and the degree and rate of weight loss when determining malnutrition levels resulting from restrictive eating disorders. Individuals with AN or AAN may engage in food restriction by limiting overall food intake, excluding certain food categories (e.g., reducing carbohydrates or desserts), or reducing the frequency of meals or snacks [28]. Despite targeted research in this area, specific evidence-based interventions to reduce restrictive eating behaviors remain lacking [29,30,31,32].

Median %En for carbohydrates was closer to the lower limit of the recommendations (45–60%), while median %En for total fat was near the middle of the RI range (20–35%En). Regarding proteins, the comparison between the observed protein intake with the AR and PRI showed an intake value at least double that recommended.

Our analysis of nutritional parameters documented eating behaviors oriented towards a reduction of the energy intake, detected by the 24 hDR, in comparison with the DRVs of the age category. It is peculiar how the weight remains substantially stable over time, from T0 onwards. The progressive reduction of the supply of nutrients may have led to a decrease in the basal metabolic rate. Determining resting energy expenditure, by using direct or indirect calorimetry, may be important in the nutritional assessment of adolescents with FEDs and how it varies over time [33].

This study has some limitations. The food survey carried out through the 24 hDR may not be consistent with the real nutritional intake. Probably, it would be necessary to evaluate the energy intake over several consecutive days to obtain a more realistic result of the energy and nutrient intake and how it varies day by day as a function of the presence or absence of binge eating and/or purging behaviors [33]. The utilization of BIA for tracking body composition introduces inherent limitations that need consideration. BIA, as employed in this research, may not be optimal for comprehensive tracking due to its known constraints, which include sensitivity to factors such as hydration status, assumptions underlying prediction methods, and variations in measurement protocols. While it provides valuable insights, its precision and accuracy may be influenced by these factors, potentially affecting the reliability of longitudinal data. Furthermore, the reliance on a single 24 hDR to assess nutritional intake poses a limitation. A single recall may not fully capture the variability in dietary patterns, potentially leading to an incomplete representation of participants’ nutritional habits. Lastly, the study’s sample size, comprising 17 children and adolescents with AAN, is relatively small. The small sample size may limit the generalizability of these findings to a broader population and could impact the statistical power to detect significant changes over time. These limitations highlight areas for caution in the interpretation of the results and underscore the importance of future research endeavors with larger samples and alternative methodologies for tracking body composition.

Nonetheless, this study also shows some strengths: it represents the largest sample of patients with AAN assessed from a nutritional point of view described in the literature so far; the included patients were assessed during three outpatient visits by adopting a standardized assessment for nutritional features, the 24 hDR, and a systematic tool to monitor changes in body composition. Future case-control studies should verify these results in larger samples.

## 5. Conclusions

This is the first study to systematically investigate the nutritional features and the body composition of a sample of children and adolescents with AAN, in a longitudinal design and adopting standardized assessments. The included individuals showed eating behaviors oriented towards low daily energy intake (with low carbohydrates and fat) and increased proteins. A longer latency before observation was correlated with a negative change in weight measures. Body composition parameters were reported, with no substantial changes across the ambulatorial evaluations.

The strengths of this study include its longitudinal nature and valuable preliminary information regarding the nutritional assessment of AAN in children and adolescents, an aspect hitherto overlooked in other previous studies. However, among the limitations, the restricted sample size and constraints associated with the use of the employed dietary assessment tool are highlighted. Additionally, it is important to note that the absence of male participants, with their specific clinical characteristics and body composition, represents an additional hurdle. In the evaluation of the encouraging results, it is believed that further investigations in this direction with larger samples may be useful to improve the understanding of the phenomenon and to assess the effect of targeted therapeutic interventions on body composition and nutritional characteristics.

## Figures and Tables

**Table 1:** Baseline sociodemographic characteristics, lifestyle behavior, clinical variables, comorbidity, family history of the disease, and pharmacological treatments in our court.

Variables	Values
Age, years	15.8 ± 1.3
Female	100%
Family history	
FED	8 (47%)
Non-FED psychopathology	8 (47%)
Clinical variables	
Duration of untreated illness, months	15.2 ± 7.7
Admission BMI, kg/m^2^	20.4 ± 2.0
Percentage BMI	99.5 ± 9.8
Weight loss between premorbid and presentation, kg	6.0 ± 5.6
Secondary amenorrhea	3 (18%)
Difficulty falling asleep	4 (24%)
Infra-hypnic awakenings	7 (41%)
FED symptoms	
Caloric restriction	17 (100%)
Purging	8 (47%)
Physical hyperactivity	11 (65%)
Binge-eating	4 (24%)
Comorbidities	
Patients with at least one comorbidity	11 (65%)
OCD	4 (24%)
MDD	6 (35%)
Anxiety disorders	9 (53%)
Self-injury	3 (18%)
Pharmacological treatment at first access	
Fluoxetine	6 (35%)
Sertraline	1 (6%)
Quetiapine	1 (6%)

Abbreviations: BMI: Body Mass Index; FED: Feeding and Eating Disorder; MDD: Major Depressive Disorder; OCD: Obsessive-Compulsive Disorder.

**Table 2:** Variation over time at admission (T0), after two months (T1), and after six months (T2), in weight expressed in kg, in the percentage of fat mass (%FM), and free fat mass (FFM) expressed in kg.

	T0	T1	T2	Statistics
Weight (kg)	54.6 ± 6.6	54.1 ± 6.0	54.3 ± 6.7	F = 0.273, *p* = 0.682
%FM	25.4 ± 5.2	25.0 ± 4.6	24.7 ± 4.3	F = 0.258, *p* = 0.775
FFM (kg)	40.5 ± 3.2	40.4 ± 3.6	40.7 ± 3.8	F = 0.59, *p* = 0.561
BMI (kg/m^2^)	20.4 ± 2.0	20.1 ± 1.8	20.2 ± 1.9	F = 0.310, *p* = 0.664

Abbreviations: BMI: Body Mass Index; %FM: percentage of fatty mass; FFM: free fatty mass.

**Table 3:** Nutritional assessment conducted through 24 h dietary recall.

Macronutrients	Mean ± SD	AR	RI	PRI	Comment
**Lipids**	28.6 ± 5.5%En	/	20–35%En	/	71% have an adequate intake
**Carbohydrates**	47.8 ± 5.5%En	/	45–60%En	/	71% have an adequate intake
**Proteins**	22.9 ± 4.5%En	/	(deducted)	/	76% have an adequate intake
**Observed protein intake, g/d**	64.0 ± 13.5	37.5 ± 4.2	/	46.7 ± 5.0	Observed intake significantly higher than both AR (*p* < 0.001) and PRI (*p* < 0.001)

Abbreviations: SD: standard deviation; AR: Average Requirement; RI: Reference Intake; PRI: Population Reference Intake; %En: percentage of Energy Intake.

## Data Availability

The data assessed and reported here can be obtained from the authors upon reasonable request and following ethical and privacy principles.
